# Supplementary material for: Ethephon-Induced Ethylene Enhances Starch Degradation and Sucrose Transport with an Interactive Abscisic Acid-Mediated Manner in Mature Leaves of Oilseed rape (Brassica napus L.)
Source: Plants (Basel). 2021 Aug 13;10(8):1670. doi: 10.3390/plants10081670 (PMC8400741; doi:10.3390/plants10081670)
Supplement: Supplementary file 1 [file plants-10-01670-s001.zip › Supplementary Table S1.pdf]

SUPPLIMENTARY TABLE S1. Primer sequences used for qRT-PCR analysis.

Gene	Accession No.	Forward sequence (5'-3')	Reverse sequence (5'-3')
Cab	AY288914	GGCAGCCCATGGTACGGATC	CCTCCITCGCTGAAGATCTGT
SAG12	XM_013821610.2	AGAGAATACCAAACCAAACCGAA	GCAACTCCCAAAATCTCAGGG
NCED3	HQ260434	GGAGTGCTTCTGCTTCCATC	TTCGAGGTTGACTTGCTCCT
PYR1	XM_013799169.2	ACCGCAGACCTACAAGCACT	CTCTCCCTCTCGAACCTGTG
SnRK2	LK937699.1	TGAAGATGAGGCTCGGTTCT	TGCCATCATATTCCTGACGA
AREB2	HE616526.1	TGCCATCATATTCCTGACGA	CACCTCTTATCCCAGGACCA
MYC2	XM013880351	ACCAAACGTCTCGAAAATGG	TGTCAACGAGCAAGAGGATG
AMY3	XM_013846160.2	GGTTACCTCCACCGACAGAA	GTTCAGACGCCCTCCAAATA
BAM1	XM_013852497.2	GAAGGTGGGGCTAAAGGTTC	GCACGCATGAAATCAGAGAA
SUT1	XM_013855840.2	GATCCTGCGGATGAGGATAA	AAGCAGCTTGTCATCCGAGT
SUT4	XM_022699588.1	AAGAAGCTTCCACGTCCAGA	TCTATGCCATTGCCAACGTA
SWEET11	XM_013871605.2	AGGTGACCCTCGATATGCTG	TCATGTAGCTGTTGCGGAAG
ACTIN	AF111812	GATTCCGTTGCCCTGAAGTA	GCGACCACCTTGATCTTCAT
