# Supplementary material for: Ethephon-Induced Ethylene Enhances Starch Degradation and Sucrose Transport with an Interactive Abscisic Acid-Mediated Manner in Mature Leaves of Oilseed rape (Brassica napus L.)
Source: Plants (Basel). 2021 Aug 13;10(8):1670. doi: 10.3390/plants10081670 (PMC8400741; doi:10.3390/plants10081670)
Supplement: Supplementary file 1 [file plants-10-01670-s001.zip › Supplementary Figures_Lee et al..pptx]

## Slide 1
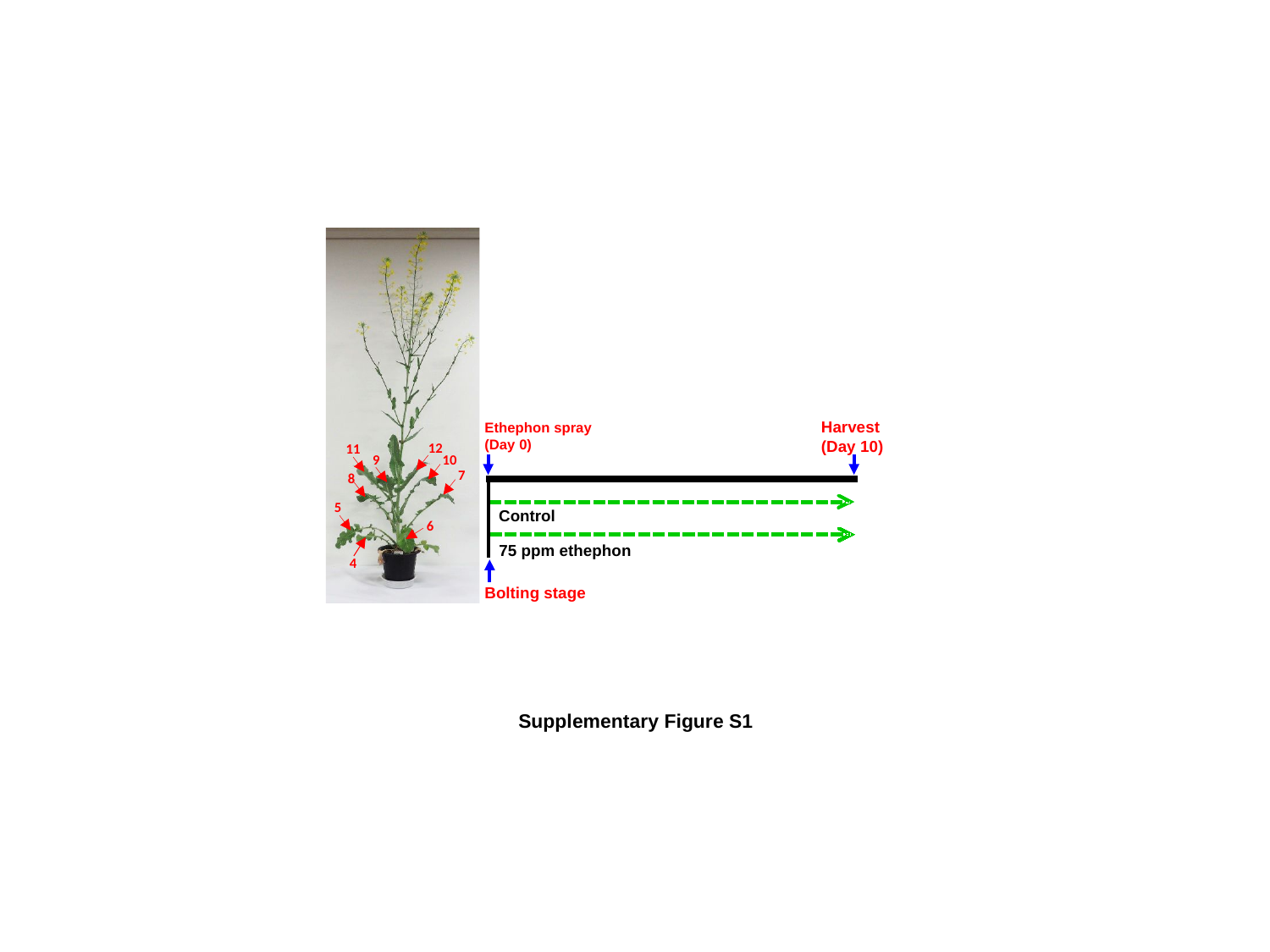

Harvest
(Day 10)
Control
Bolting stage
Ethephon spray
(Day 0)
12
11
9
10
7
8
5
6
75 ppm ethephon
4
Supplementary Figure S1

## Slide 2
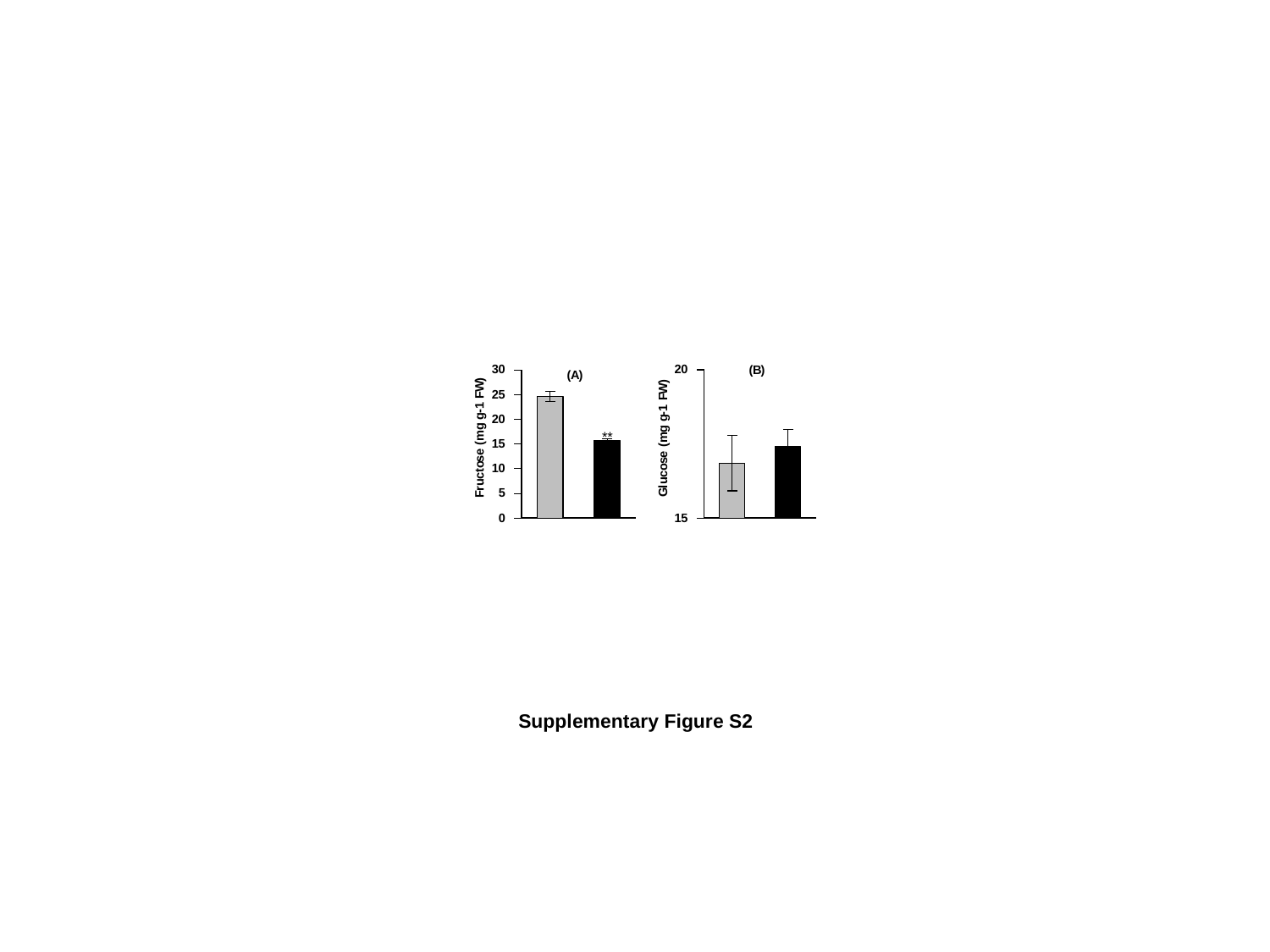

### Chart
| Category | Fructose (mg g-1 FW) |
|---|---|
| Control | 24.68157695434822 |
| Ethephon | 15.691371053372881 |
### Chart
| Category | glucose |
|---|---|
| Control | 16.855482872186393 |
| Ethephon | 17.41637338451269 |Supplementary Figure S2
